# Nutritional Status in a Contemporary Urban Population-Based Cohort: Prevalence, Clinical Associations and Long-Term Health Outcomes

**DOI:** 10.3390/nu18193228

**Published:** 2026-09-30

**Authors:** Katja Sibylle Mühlberg, Sabine Steiner, Naomi Elbaranes, Zoe Vente, Markus Scholz, Dierk Scheinert, Sophie Brunner-Ziegler

**Affiliations:** 1 Department of Angiology, University Hospital Leipzig, 04109 Leipzig, Germany; katja.muehlberg@medizin.uni-leipzig.de (K.S.M.);; 2 Helmholtz Institute for Metabolic, Obesity and Vascular Research (HI-MAG), Helmholtz Center Munich, University of Leipzig and University Hospital Leipzig, 04103 Leipzig, Germany; sabine.m.steiner@meduniwien.ac.at; 3 Division of Angiology, Department of Internal Medicine II, Medical University of Vienna, 1090 Vienna, Austria; naomi.elbaranes@meduniwien.ac.at; 4 Institute for Medical Informatics, Statistics, and Epidemiology (IMISE), University of Leipzig, 04107 Leipzig, Germany

**Keywords:** nutritional deficiencies, nutritional scores, prognostic nutritional index (PNI), LIFE-Adult study, long-term survival

## Abstract

Background: Nutritional deficiencies are associated with poor prognosis in a wide range of illnesses. However, the prognostic impact of poor nutritional status with respect to long-term all-cause mortality in the general non-diseased population is not well described. Objectives: This study sought to report the prevalence, clinical associations, and prognostic consequences of poor nutritional status in a contemporary urban population in Germany. Methods: A cohort of 8398 participants from the LIFE-Adult Study was enrolled. Four objective nutritional scores, the Prognostic Nutritional Index (PNI), the Controlling Nutritional Status Score (CONUT), the Nutritional Risk Index (NRI), and the Triglyceride Cholesterol Body Weight Index (TCBI), were calculated at baseline, and participants’ nutritional status was classified according to score tertiles. The relationship between nutritional status and common risk factors as well as major diseases was assessed. In addition, the association between poor nutritional status at baseline and the risk of all-cause mortality during a seven-year follow-up period was examined. Results: According to the WHO definition of obesity, the LIFE-Adult Study cohort was, on average, overweight, with almost two-thirds of participants (67%) having a BMI ≥ 25 and only 1% being underweight. However, poor nutritional status was not associated with a specific BMI category. Higher age, female sex, cancer, and chronic kidney disease were more common, whereas active smoking and hypercholesterolemia were less prevalent among participants in the lowest PNI tertile (*p* < 0.01). In the overall population, the cumulative incidence of all-cause mortality was significantly higher among participants in the lowest PNI, NRI, and TCBI tertiles and the highest CONUT category (log-rank test, *p* < 0.01 for PNI, CONUT, and NRI, and *p* < 0.04 for TCBI, respectively). After adjustment for confounding factors, the lowest PNI and NRI tertiles and the highest and medium CONUT categories remained independently and significantly associated with an increased risk of all-cause mortality (all *p* < 0.01) in multivariable Cox regression analyses. TCBI was not associated with all-cause mortality in any regression model. Conclusions: Poor nutritional status is associated with increased mortality in the general population. Clinical trials are needed to prospectively evaluate the efficacy of nutritional interventions on health outcomes.

## 1. Introduction

By definition, malnutrition (poor nutritional status/nutritional deficiency) is characterized by an insufficient supply or impaired assimilation of essential nutrients, leading to a lack of energy and protein, loss of muscle mass [1,2] and impairment of bodily functions [3,4].

In recent years, nutritional deficiencies have been classified by the World Health Organization (WHO) as a major threat to public health worldwide, with a detrimental impact on overall morbidity, mortality, and quality of life, resulting in a significant individual, social, and economic burden [1,4].

There is consensus that poor nutritional status is highly prevalent among patients with cardiovascular risk factors or chronic conditions such as cancer and end-stage renal disease and that it is associated with decreased physical, cognitive, and immune function as well as a worsened health prognosis [5,6,7,8], including an increased rate of premature death from all causes [9,10].

Because the causes of nutritional deficiencies are usually multifactorial and complex, a comprehensive and reproducible evaluation of these conditions is challenging. According to the recommendations of the European Society for Clinical Nutrition and Metabolism, nutritional deficiencies should initially be identified using validated screening tools. Subsequent nutritional assessment may incorporate nutritional scoring systems calculated from anthropometric and blood parameters [3]. In this regard, the Prognostic Nutritional Index (PNI) [11], the Controlling Nutritional Status score (CONUT) [12], the Nutritional Risk Index (NRI) [13] and the Triglyceride Cholesterol Body Weight Index (TCBI) [14] have gained international acceptance for generating a meaningful picture of an individual’s nutritional status. As such instruments, they have already been used in numerous investigations across a wide range of clinical conditions, e.g., coronary artery disease, heart failure, chronic kidney disease and cancer, with the majority of studies demonstrating a significant association between poor nutritional status and adverse health outcomes [6,8,13,14,15,16]. However, although these indices were developed to identify patients at nutritional risk, their individual components may also be influenced by factors unrelated to nutritional intake, including underlying disease-specific pathophysiology or disease severity. Consequently, the same score or threshold may not have identical clinical implications across different patient populations. Therefore, predefined cut-off values should be interpreted with caution and, where appropriate, adapted to the disease-specific characteristics of the population under investigation.

In contrast, less attention has been paid to identifying potential nutritional deficiencies in the general population of industrialized countries, as these populations are often assumed to live in a universal state of abundance, including an oversupply of food. Consequently, only a limited amount of data is available using nutritional variables to systematically examine the prevalence, determinants, and consequences of poor nutritional status in non-diseased individuals who are not at risk of starvation and for whom inadequate nutrient intake due to illness and/or food shortage is unlikely. In particular, predictive models investigating the association between nutritional deficiencies and adverse long-term survival outcomes in these populations are lacking.

To successfully combat nutritional deficiencies, evidence-based knowledge is needed regarding the modifiable determinants of this complex condition on which physicians and affected individuals themselves can act. However, as long as nutritional screening is not routinely performed in the general population, individuals at risk may remain undetected and untreated [17,18].

Our study aimed to evaluate the value of four different nutritional scoring systems for assessing the prevalence, determinants, and prognostic consequences of poor nutritional status in a cohort of the contemporary LIFE-Adult Study population from Leipzig, Germany. In particular, the focus was on the influence of poor nutritional status on long-term survival in the general population in order to provide decision-makers with a basis for implementing screening and early detection measures targeting nutritional deficiencies.

## 2. Methods

### 2.1. Study Population and Recruitment

The LIFE-Adult Study recruited an age- and sex-stratified random sample of 10,000 residents of the city of Leipzig between August 2011 and November 2014. The detailed study protocol and objectives have been published previously [19]. A description of the study population can be found elsewhere [20]. Individuals interested in participating were invited to attend an appointment at the Leipzig Research Centre for Civilization Diseases. Written informed consent was obtained from all participants before enrolment. The study design was reviewed and approved by the Ethics Committee of the Medical Faculty of the University of Leipzig (LIFE-Adult: Reg. No. 263-2009-14122009 dated 8 November 2010), and the Saxony Data Protection Officer was responsible for data protection and safety.

Among the entire study cohort, nutritional status based on the PNI could be assessed in 8398 participants, who were included in the present analysis. Participants with incomplete records for variables required to calculate the nutritional score were excluded from the present analysis.

### 2.2. Collection of Baseline and Follow-Up Information

The assessment program comprised physical and medical examinations as well as computer-assisted personal interviews and self-administered questionnaires. At baseline, sociodemographic information, including age, sex, and socioeconomic status, as well as medical history, including cardiovascular risk factors (smoking status, arterial hypertension, diabetes, hypercholesterolemia, and obesity), comorbidities, and current medications, were recorded for all participants. In addition, the nutritional status of participants was assessed using four nutritional scoring systems.

For the main LIFE-Adult Study, longitudinal follow-up started in 2017 and ended in 2021. Information on vital status was obtained through queries of the population registry.

### 2.3. Definitions and Calculations of Covariates and Outcomes

Participants were stratified into three socioeconomic status categories: low, medium, and high. Body mass index (BMI) was calculated using the following formula: body weight (kg)/height^2^ (m^2^). According to the WHO, adult weight classes were defined as underweight for a BMI < 18.5 and normal (healthy) weight for a BMI ≥ 18.5 and <25.0. Pre-obesity was classified between ≥25.0 and <30.0, while obesity starts at a BMI ≥ 30.0. Smoking status was categorized as never, former, or current smoker. Coronary artery disease (CAD) was defined as a history of angina pectoris, myocardial infarction, and/or coronary artery revascularization by means of surgical or endovascular intervention. Cerebrovascular disease was defined as the presence of hemodynamically significant atherosclerotic plaques in the supra-aortic carotid arteries and/or a history of stroke and/or carotid artery revascularization by means of surgical intervention. Peripheral artery disease (PAD) was defined as a history of intermittent claudication and/or peripheral artery revascularization by means of surgical or endovascular intervention and/or an ankle-brachial index (ABI) ≤ 0.9. The composite endpoint of cardiovascular disease was defined as a history of CAD and/or cerebrovascular disease and/or PAD. Arterial hypertension was considered present if participants were taking antihypertensive medication, had a mean systolic blood pressure > 140 mmHg, and/or mean diastolic blood pressure > 90 mmHg. Diabetes was defined as glycated hemoglobin (HbA1c) ≥ 6.5% and/or the use of antidiabetic medication. Hypercholesterolemia was defined as total cholesterol ≥ 200 mg/dL and/or the use of lipid-lowering medication. Chronic obstructive pulmonary disease was defined according to the Global Initiative for Chronic Obstructive Lung Disease (GOLD) criteria as persistent respiratory symptoms and airflow limitation. Chronic kidney disease was defined as chronic impairment of kidney function, indicated by an estimated glomerular filtration rate (eGFR) < 60 mL/min/1.73 m^2^ persisting for at least three months. As part of the collection of baseline data, participants were also asked about the diagnosis of any malignancy. Engaging in regular physical activity was measured by the self-reported International Physical Activity Questionnaire, and ratings of 2.9, 4.0, and 7.0 MET-minutes per week were assigned for light-intensity, moderate-intensity, and vigorous-intensity activities [21].

The primary long-term outcome was all-cause mortality during follow-up.

### 2.4. Nutritional Scores

Nutritional status was assessed using four comprehensive nutritional assessment tools: the Prognostic Nutritional Index (PNI), the Controlling Nutritional Status (CONUT) score, the Nutritional Risk Index (NRI), and the Triglyceride Cholesterol Body Weight Index (TCBI).

The formulas used to calculate these scores include objectively measurable parameters (serum albumin, total cholesterol, lymphocyte count, and body weight). As a result, these scores provide efficient and simple screening tools for protein-energy malnutrition and are frequently used in clinical nutritional research [5,6,8,11,12,13,14,15,16].

Conventionally, low PNI, NRI, and TCBI values and/or a high CONUT score indicate nutritional deficiency, whereas medium to high PNI, NRI, and TCBI values and/or a low CONUT score indicate normal to good nutritional status.

The following formulas were used:

PNI = serum albumin (g/L) + 5 × total lymphocyte count (10^9^/L) [11].

NRI = [1.489 × serum albumin (g/L)] + [41.7 × weight (kg)/ideal body weight (kg)], whereby ideal body weight was calculated as 22 × height^2^ because of its validity. The weight-to-ideal body weight ratio was set to 1 if actual body weight exceeded ideal body weight [13].

TCBI = serum triglycerides (mg/dL) × total cholesterol (mg/dL) × body weight (kg)/1000 [14].

The CONUT score was calculated as the sum of predefined scores assigned to serum albumin, total cholesterol, and lymphocyte count values. Albumin values ≥ 35 g/L, 30–34.9 g/L, 25–29.9 g/L, and ≤24.9 g/L corresponded to scores of 0, 2, 4, and 6 points, respectively. Lymphocyte counts ≥ 1.6 × 10^9^/L, 1.2–1.59 × 10^9^/L, 0.8–1.19 × 10^9^/L, and <0.8 × 10^9^/L corresponded to scores of 0, 1, 2, and 3 points, respectively. Likewise, total cholesterol values ≥ 180 mg/dL, 140–179 mg/dL, 100–139 mg/dL, and ≤99 mg/dL corresponded to scores of 0, 1, 2, and 3 points, respectively [12].

For the description of baseline characteristics, the PNI was selected to categorize participants into three groups according to their nutritional status. The selection of the PNI was based on two main considerations. First, previous studies have demonstrated high prognostic accuracy of the PNI for identifying clinically relevant nutritional deficiencies. Second, the PNI showed at least a weak correlation with the other nutritional assessment scores used in our study [8,11].

### 2.5. Statistical Analysis

Data analysis was conducted using SPSS version 29.0 (IBM Corp., Armonk, NY, USA). Continuous variables are presented as means ± standard deviation, and categorical variables as frequencies and percentages. Baseline characteristics were compared across the three PNI groups using one-way analysis of variance (ANOVA), independent-samples t-tests, or chi-square tests, as appropriate.

Correlations between the PNI, NRI, TCBI, and CONUT scores were assessed using Spearman’s rank correlation coefficient (ρ), because of the non-normal distribution of the variables and, in particular, the marked skewness of the CONUT score.

Cox proportional hazard regression analyses were performed for time-to-death outcomes, and differences between tertiles/categories of the nutritional scores were assessed using log-rank tests. To evaluate the predictive value of the four nutritional scores for all-cause mortality, three different Cox proportional hazard models were applied to calculate crude and adjusted nutrition deficiency-related mortality risks. Model 1 represented the crude analysis, only including the nutritional scores. Model 2 additionally adjusted for age and sex to allow the incremental effect of additional covariates to be evaluated in the subsequent model. The adjustment strategy for Model 3 was defined based on an a priori hypothesized causal structure and established epidemiological considerations: by creating a directed acyclic graph, we mapped the hypothesized causal relationships between demographic, lifestyle, socioeconomic, and pre-existing clinical factors that could plausibly influence both baseline nutritional status and subsequent mortality, while avoiding adjustment for variables that may represent mediators or colliders. Based on this framework, age, sex, socioeconomic status, smoking, physical activity level, cardiovascular disease, chronic obstructive pulmonary disease, and cancer were included in the multivariable model 3. 

To further evaluate whether the prognostic relevance of values within the lowest score tertiles (PNI NRI, TCBI) and for the highest score category (CONUT) differed between subgroups defined by the covariates included in Model 3, we performed interaction analyses in the overall study population. Statistical significance was defined as *p* < 0.05.

## 3. Results

### 3.1. Baseline Characteristics

The initial cohort consisted of 10,000 participants. Of these, 8398 participants had complete data on serum albumin and absolute lymphocyte count and were therefore eligible for calculation of the prognostic nutritional index (PNI). The remaining 1602 participants were excluded from the present analysis because of missing data for one or more of these parameters. With respect to age, sex, and socioeconomic status, exploratory comparisons revealed no substantial differences between participants included in the final analytical cohort and those excluded due to missing data on serum albumin and/or absolute lymphocyte count.

The baseline characteristics of all participants eligible for analysis (*n* = 8398) are displayed in the left column of Table 1.

**Figure 1:**
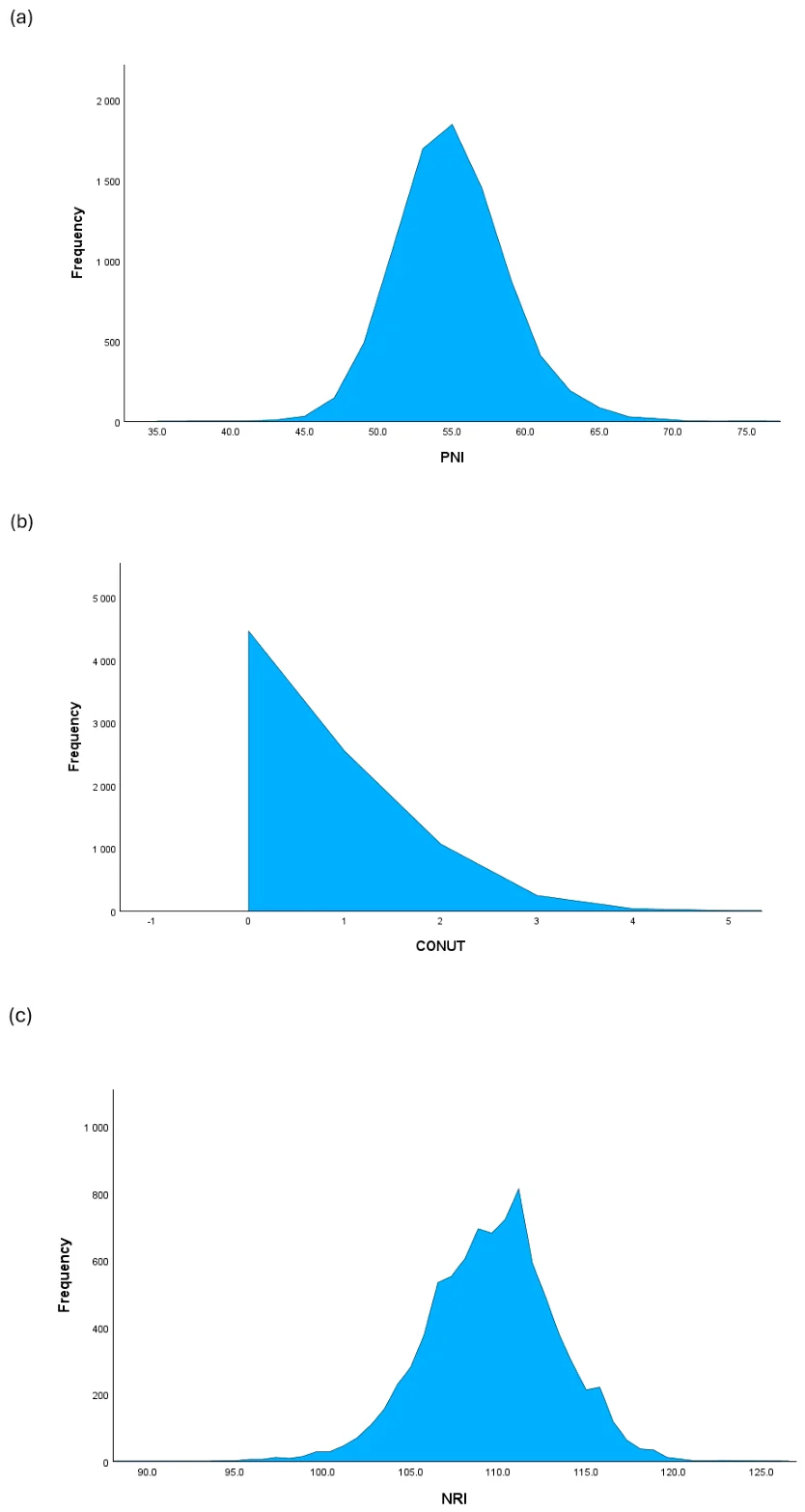

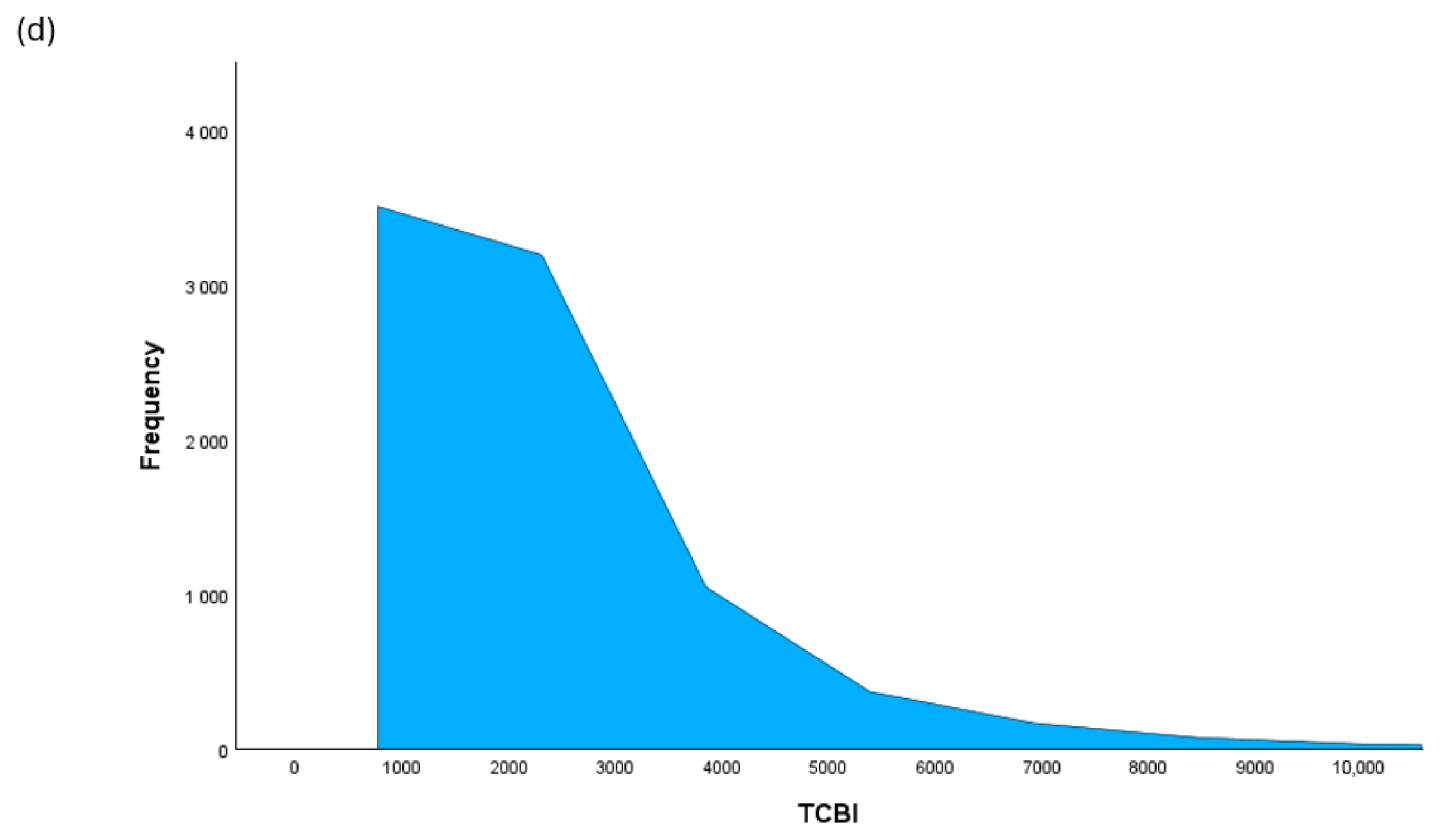
Population distribution of the four nutritional scores: (**a**) PNI, (**b**) CONUT, (**c**) NRI, and (**d**) TCBI.

Distributions of the nutritional scores PNI, NRI, TCBI, and CONUT are shown in Figure 1. PNI and NRI were approximately normally distributed, whereas TCBI and the CONUT score showed a strongly right-skewed distribution. A weak to moderate positive correlation at the 0.001 level (two-tailed) was observed among the PNI, NRI, and TCBI scores (r^s^ = 0.63 for PNI vs. NRI, r^s^ = 0.19 for PNI vs. TCBI, and r^s^ = 0.15 for NRI vs. TCBI, respectively). In contrast, the CONUT score was inversely correlated with all three scores (r^s^ = −0.56 with PNI, r^s^ = −0.08 with NRI, and r^s^ = −0.26 with TCBI; *p* < 0.001 for all, respectively).

For the purpose of the present analysis, participants were categorized according to their nutritional status using tertiles of the PNI, NRI, and TCBI score distributions in the overall study population. This approach was chosen instead of applying predefined cut-off values, as fixed thresholds may not adequately account for differences in disease-specific pathophysiology and clinical characteristics. The cut-off values were 53.3 and 56.3 for the PNI, 108.2 and 111.2 for the NRI, and 1336.9 and 2352.4 for the TCBI. Owing to the strongly right-skewed distribution of the CONUT score, no tertile division was performed; instead, cut-off values were set at 0 and ≥3.

To additionally present demographic information of our study cohort according to nutritional status, we chose PNI tertiles because this score is most commonly used in comparable studies addressing similar research questions and because of its correlation with the three other scoring systems described above. For the purpose of the present study, “nutritional deficiency”, also referred to as “poorest nutritional status”, pertained to the respective lowest score tertiles of the PNI, NRI and TCBI, and the highest category of the CONUT; for the PNI, “nutritional deficiency” was specifically defined as values within the lowest PNI tertile (PNI < 53.3).

The mean BMI of the entire study population was 27.5 (±4.8). Thus, according to the WHO definition, the study cohort was, on average, overweight, with almost two-thirds of participants (67%) having a BMI ≥ 25. One quarter of the sample was obese (BMI ≥ 30), and only one percent was underweight (BMI < 18.5). BMI did not differ significantly between the three PNI tertiles. The proportion of participants older than 65 years was higher than in the other two PNI groups. Female sex, history of cancer, and chronic kidney disease were more common among participants within the lowest PNI tertile (*p* < 0.001), whereas active smoking, hypercholesterolemia, diabetes, and physical activity at light levels were least prevalent among those belonging to the lowest PNI group (*p* < 0.01).

With the exception of BMI, all numerical variables presented in Table 1 differed significantly between participants with and without nutritional deficiency. Participants assigned to the lowest PNI tertile had lower GFR values and lower systolic and diastolic blood pressure than those assigned to the middle and highest PNI tertiles. Furthermore, serum levels of albumin, total cholesterol, and triglycerides, as well as lymphocyte counts, were lower in nutrition-deficient individuals.

### 3.2. Mortality Risk

**Figure 2:**
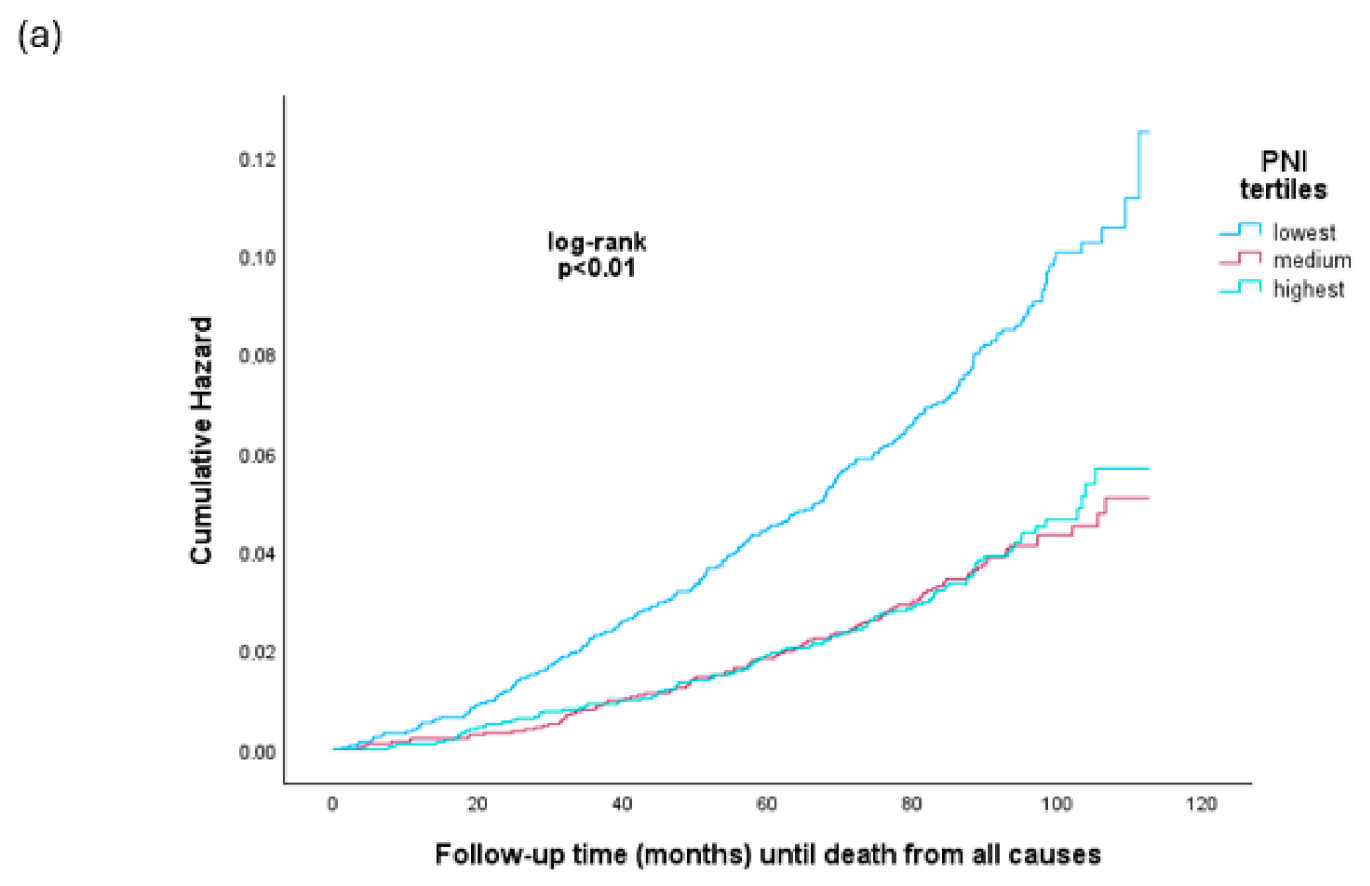

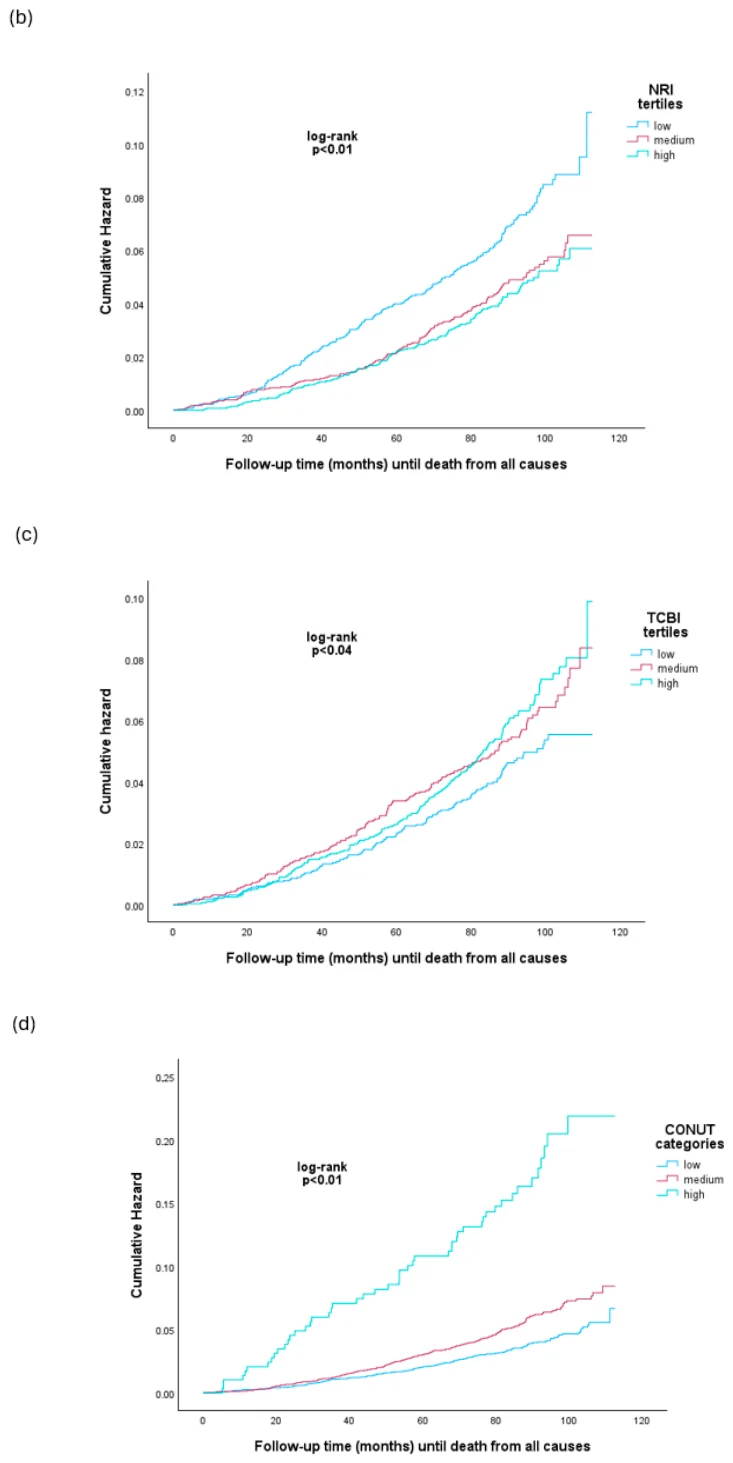
Cox proportional hazards regression curves for all-cause mortality based on the four nutritional scores: (**a**) PNI, (**b**) CONUT, (**c**) NRI and (**d**) TCBI.

During a median follow-up period of 90.8 months (IQR: 0–112.6 months), 444 participants (5.3%) died from various causes, whereas 7954 survived. Figure 2 illustrates curves of Cox proportional hazards regression analysis for the tertiles of each of the four nutritional scores, revealing a significantly increased incidence of all-cause mortality among participants with a poor nutritional status: within the overall population, the cumulative incidence of all-cause mortality was higher among participants with the lowest PNI, NRI, and TCBI tertiles and the highest CONUT category, compared with the other tertiles/categories, respectively. For TCBI, the separation of Kaplan–Meier survival curves for all-cause mortality was less pronounced, with weaker statistical evidence for between-group differences (log-rank test, *p* = 0.04), whereas the other three nutritional scores showed stronger evidence of differences in survival (log-rank test, *p* < 0.01 for all three scores).

### 3.3. Multivariate Interaction Analyses

Table 2 presents the results of the regression analyses assessing the association between the four nutritional scores and mortality. For each score, mortality risk was compared across the respective tertiles/categories, using the tertile/category corresponding to the best nutritional status as the reference category. Three Cox proportional hazards models were fitted to assess these associations at different levels of adjustment. Model 1 was unadjusted (univariate analysis), and Model 2 adjusted for age and sex. Model 3 adjusted for age, sex, socioeconomic status, smoking, physical activity, cardiovascular disease, chronic obstructive pulmonary disease, cancer, diabetes, and chronic kidney disease. These models allowed the associations between nutritional status and all-cause mortality to be examined both before and after adjustment for potential confounders. In both univariate and multivariate Cox proportional hazards analyses, participants with score values within the lowest PNI and NRI tertiles had an increased risk of all-cause mortality compared with those in the highest tertiles, whereas participants with score values within the highest CONUT category showed an increased risk of all-cause mortality compared with those in the lowest CONUT category. In addition, intermediate CONUT scores were associated with an increased incidence of all-cause mortality in comparison to values within the lowest category of this score in the univariate analysis and in Model 3. However, this latter effect was no longer statistically significant in Model 2. TCBI was not associated with all-cause mortality in any of the three models.

Interaction analyses, assessing whether the predictive value of “poorest nutritional status” for all-cause mortality varied according to relevant population characteristic variables, found no evidence of effect modification for any of the variables included in the adjustment of Model 3.

## 4. Discussion

The present study characterized nutritional status using well-established nutritional scores that can be readily calculated from routine laboratory data and its associations with demographic and health-related factors in a population-based urban cohort from eastern Germany using established nutritional scores. In addition, we evaluated the impact of poor nutritional status on long-term all-cause mortality.

### 4.1. Prevalence and Determinants of Poor Nutritional Status

An association was observed between the Prognostic Nutritional Index (PNI) and the other nutritional scores applied in this study. Although the cohort was overweight on average according to World Health Organization criteria, weight classification based on BMI did not show significant differences across PNI tertiles.

An American cohort study with a comparable age distribution reported lower mean nutritional scores than those observed in our study [5]. Our findings differ from previous reports describing higher BMI values among participants in the lowest PNI tertile [22], as well as from studies reporting a higher prevalence of nutritional deficiencies among underweight patients [23]. In our cohort, however, BMI did not differ significantly across PNI tertiles. These discrepancies may reflect substantial heterogeneity in study populations, healthcare settings and nutritional assessment methods, and therefore, cross-study comparisons should be interpreted with caution. Nevertheless, taken together, findings suggest that, at the population level, BMI alone is insufficient to adequately capture differences in nutritional status. Rather, BMI and nutritional status should be considered related but distinct dimensions of health, with nutritional assessment providing complementary information beyond conventional anthropometric measures.

In the present analysis, poor nutritional status was primarily associated with older age, female sex, a history of cancer, and presence of chronic kidney disease. In contrast, no association was found with chronic obstructive pulmonary disease, rheumatologic disease, or established cardiovascular disease. Unexpectedly, active smoking, elevated blood pressure, hypercholesterolemia, and diabetes showed an even inverse association with the risk of poor nutritional status. Regarding regular engagement in physical activity, differences between the nutritional score tertiles were most pronounced for light levels of physical activity, showing individuals in the lowest tertile of the PNI substantially less active and those in the highest PNI tertile more physically active at light physical activity levels than their corresponding population means.

The increase in prevalence of nutritional deficiency with advancing age corroborates previous evidence that age-related impairments in food intake and nutrient absorption contribute to nutritional deficiencies [24]. Nevertheless, female participants of our study cohort had a higher prevalence of nutritional deficiency despite being younger. This may partly reflect sex-related differences in the components of nutritional scores. As an example, men, as described in the literature and as demonstrated in our study, tend to have higher absolute values of serum albumin. As a result, a well-nourished man appears to have a better nutrition state according to the calculated PNI than a woman. Further, women with the same level of physical fitness have less muscle mass than men, which might automatically influence scores that include body weight in their calculations, such as the NRI or the TCBI [24].

We found no clear relationship between low socioeconomic status and poor nutritional status, which contrasts with several international studies linking lower education, unemployment, or poverty to nutritional deficiency and/or poorer dietary quality [25,26]. However, the recruitment strategy of the LIFE-Adult Cohort may have introduced selection bias toward healthier and better educated individuals with greater healthcare utilization, potentially leading to an underestimation of the prevalence of nutritional deficiencies and thereby limited generalizability [20].

Also, due to the mandatory health insurance requirement, access to healthcare services—and thus to nutritional medical education—is available to everyone in Germany, including low-income population groups. As a result, severe nutritional deficiencies are likely to occur less frequently in Germany than in countries without free access to healthcare [26,27].

Our results confirm previous reports identifying a history of cancer as a major determinant of poor nutritional status [28,29]. This association appears consistent across different study populations and assessment methods. By contrast, we did not observe an association between poor nutritional status and cardiovascular disease, unlike some earlier studies [5]. Possible explanations include the generally higher PNI values in our cohort than in the U.S. comparison study and that our definition of poor nutritional status, referring to the lowest third of all score values, is not equivalent to severe poor nutritional status as defined by others based on established cut-off values. In addition, patients with cardiovascular disease in Germany may receive more frequent medical care and nutritional counseling, which could mitigate the risk of nutritional deficiency.

In fact, we even observed a negative association between selected cardiovascular risk factors, such as active smoking, elevated blood pressure, diabetes, and hypercholesterolemia, and the likelihood of being classified within the lowest tertile of nutritional status, despite the fact that the proportion of participants receiving cardiovascular protective therapy, at least with respect to arterial hypertension and hypercholesterolemia, was roughly equal across the different nutrition states.

Nevertheless, this does not imply that tobacco use and presence of arterial hypertension or hypercholesterolemia are synonymous with, or indicative of, a better nutritional status.

### 4.2. Association Between Nutritional Scores and All-Cause Mortality

In accordance with Fan et al., lower PNI and NRI values and higher CONUT scores were independently associated with increased long-term all-cause mortality in the entire sample of our study population [5]. Interaction analyses suggested that the association between nutritional deficiencies and mortality risk did not differ according to the presence of chronic diseases, including diabetes, cardiovascular disease, cancer, COPD, and chronic kidney disease. In addition, we found no evidence of effect modification by sex, age, or lifestyle factors such as smoking and physical activity. These findings suggest that the association between poorer nutritional status and mortality may be broadly applicable across the general population and thereby might extend beyond individuals with specific underlying health conditions, as already evidenced by previous studies [6,7,8,9,15,16]. However, as long as nutritional screening is still not performed as a routine measure in the general population, people at risk may generally remain undetected and untreated [18].

Based on the current literature speculations, the negative impact of poor nutritional status is suggested to be related to a multifactorial organic disbalance, including increased oxidative stress and blood viscosity, impaired neurohormonal function, systemic inflammation, and weakened physiological reserve [8].

Given the observational nature of this analysis, these findings do not establish causality or support the use of PNI as a standalone clinical decision-making tool, as poor nutritional status may also reflect underlying disease severity and complexity. Nevertheless, because PNI is based on routinely available clinical parameters, it may serve as an accessible marker to identify individuals who may benefit from closer risk assessment and targeted preventive strategies. These findings underscore the potential importance of early identification and management of nutritional deficiencies. Prospective studies are warranted to determine whether incorporating nutritional scores into established risk stratification approaches can improve clinical decision-making and preventive care. In the context of rising healthcare costs, greater awareness among healthcare professionals, patients, and policymakers regarding the screening, early detection, and treatment of nutritional deficiencies may be essential to addressing this growing public health challenge.

## 5. Conclusions

In summary, in this population-based cohort, overweight and obesity remained common. Notably, BMI did not differ significantly across PNI tertiles, suggesting that nutritional status cannot be adequately characterized by BMI alone. Although poor nutritional status appeared to be more prevalent among individuals of older age, female sex, and those with a history of cancer or chronic kidney disease, its association with adverse long-term survival remained after adjustment for sex, age, lifestyle factors, cardiovascular risk factors, and chronic comorbidities.

These findings support the broader integration of standardized nutritional assessment into routine clinical practice and population-based early detection and preventive strategies. Because nutritional screening is not routinely performed in the general population, individuals at risk may remain undetected. Given the growing burden of chronic disease and associated healthcare costs, greater awareness of nutritional screening, early identification, and targeted intervention may be warranted.

## 6. Strengths and Limitations of the Study

### 6.1. Strengths

A major strength is that the sample studied in this investigation was a general population-based cohort with a high number of cases, a wide age spectrum, and an almost balanced sex proportion and socioeconomic status distribution.

### 6.2. Weaknesses with Respect to the Nutritional Scores

Comparing individual investigations on nutritional deficiencies is generally challenging, as the definitions and diagnostic criteria for a poor nutrition status can vary significantly between studies. Moreover, there are crucial differences regarding the selection of study participants based on age, health status, and socioeconomic status, and also regarding the national healthcare system of the respective study countries [5,23]. In this context, it is also noteworthy that Naber et al. demonstrated a variance of around 40% to 60% in the detection of malnutrition depending on the measurement instrument used [30].

The nutritional scores used for the present investigation have certain limitations. For all scores, dilution effects, such as excessive fluid intake, can lead to a distortion of the calculated values. Furthermore, the scores do not detect deficiencies in the field of micronutrition, as minerals, vitamins, or hormones are not included. Additionally, irregular food intake, which can also be an important cause of a poor nutrition status [28] is not accounted for by the scores used in this study. 

In the case of the PNI, an increase in the lymphocyte count, which might occur in infections, hematopoietic proliferative diseases, or even during pregnancy [29], may falsely suggest a better nutritional state. Similarly, albumin might be strongly influenced by subclinical inflammation and disease.

The CONUT score, which incorporates the influence of total cholesterol on nutritional state, may be overestimated due to lipid-lowering drug use in patients with hypercholesterolemia. Also, a lipid-reduced diet might mask a truly elevated cholesterol level, resulting in an erroneously higher, and therefore apparently better, CONUT score. In addition, compared with the other three scores that use continuous variables, the CONUT score formula treats its elements as categorical variables, which might exacerbate loss of information and might limit its suitability as a long-term indicator.

The NRI uses the ratio of body weight to ideal body weight, which better reflects the deviation of the malnourished population from participants with normal BMI. However, because weight may change substantially during long-term follow-up, the predictive value of the NRI might be limited in the general population. Another limitation is the categorization of continuous metrics, which may result in loss of information and reduced variability compared with analyses using the original continuous measures. Categorization also imposes artificial boundaries on an underlying continuum and assumes that individuals within the same category are sufficiently similar, despite potentially differing substantially in their underlying continuous values. This may affect the interpretation of the findings and potentially obscure associations that would be detectable when using the measures continuously. Future studies should consider retaining continuous measures where appropriate and cross-validating findings using continuous specifications to assess the robustness of the observed associations.

### 6.3. General Weaknesses

First, the observational nature of the analysis precludes any inference regarding a causal relationship between nutritional status and the outcome. Recommendations regarding the use of PNI in preventive healthcare should therefore be interpreted with caution. For the classification of obesity, we relied on BMI, as measures of body fat distribution were not available for a sufficiently large subsample of the study population. In addition, as is common in population-based studies, the LIFE-Adult Study has been shown to be subject to selection bias, with participants tending to be healthier and more health-conscious and to have a higher socioeconomic status than nonparticipants [31]. Enzenbacher et al. also reported that, compared with nonparticipants, study participants were less likely to be older women and more likely to be married, employed, and current nonsmokers [31].

These characteristics may limit the external validity of our findings. In particular, the underrepresentation of individuals with lower socioeconomic status, less healthy lifestyles, and poorer health may result in an underestimation of the prevalence of major health conditions and nutritional deficiencies in the general population.

## Figures and Tables

**Table 1:** Characteristics of the total study sample, stratified by PNI tertiles (PNI score was available in a total of 8398 participants).

*n*/% or Mean (SD)	Total *n* = 8398	Lowest PNI Tertile *n* = 2842	Medium PNI Tertile *n* = 2773	Highest PNI Tertile *n* = 2783	Asymptomatic 2-Sided Significance (Significance btw Groups)
Demographic characteristics					
Sex					<0.001
Male	4029 (48)	1297 (46)	1302 (47)	1430 (51)	
Female	4369 (52)	1545 (54)	1471 (53)	1353 (49)	
Age group					<0.001
<50	2378 (28)	732 (26)	799 (29)	847 (30)	
50–59.9	2070 (25)	594 (21)	723 (26)	753 (27)	
60–69.9	2250 (27)	759 (27)	723 (26)	768 (28)	
≥70	1700 (20)	757 (27)	528 (19)	415 (15)	
Socioeconomic status					0.039
High	175 (20)	593 (21)	561 (20)	521 (19)	
Middle	5059 (60)	1686 (60)	1700 (62)	1673 (60)	
Low	1647 (20)	556 (20)	505 (18)	585 (21)	
Anthroprometric parameters					
Body mass index	27.5 (4.8)	27.4 (5.0)	27.3 (4.7)	27.7 (4.7)	0.27
Weight group					0.002
Under weight	44 (1)	13 (1)	14 (1)	17 (1)	
Normal weight	2728 (33)	970 (34)	940 (34)	818 (29)	
Pre-obesity (BMI ≥ 25–29)	3488 (42)	1150 (41)	1150 (42)	1188 (43)	
Obesity (BMI ≥ 30)	2119 (25)	696 (25)	668 (24)	755 (27)	
Lifestyle factors					
Smoking status					<0.001
Never smoking	3864(48)	1394 (52)	1308 (49)	1162 (43)	
Former smoking	2427 (30)	876 (33)	820 (31)	731 (27)	
Active smoking	1752 (22)	419 (16)	540 (20)	793 (30)	
IPAQ classification of PA engagement					<0.012
Light physical activity levels	868 (13)	256 (11)	281 (12)	331 (14)	
Moderate physical activity levels	1954 (28)	657 (29)	675 (29)	622 (27)	
Vigorous physical activity levels	4098 (59)	1385 (60)	1372 (59)	1341 (59)	
Cardiovascular risk factors					
Arterial hypertension	4905 (58)	1659 (58)	1585 (57)	1661 (58)	0.161
Blood pressure systolic (mm Hg)	128.9 (170)	128.1 (17)	128.7 (17)	130.0 (17)	<0.01
Blood pressure diastolic (mm Hg)	75.6 (10)	74.6 (10)	75.6 (10)	76.6 (10)	<0.01
hypercholesterolemia	5587 (67)	1703 (60)	1883 (68)	2001 (72)	<0.001
Diabetes	950 (11)	300 (11)	298 (11)	352 (13)	0.024
Ccomorbidities					
Cardiovascular diseases	1963 (23)	665 (23)	630 (23)	668 (24)	0.527
Coronary heart disease	1383 (17)	470 (17)	440 (16)	473 (17)	0.522
Peripheral artery disease	618 (7)	200 (7)	207 (8)	211 (8)	0.712
Cerebral artery disease	232 (3)	90 (3)	63 (2)	79 (3)	0.118
Congestive heart failure	157 (2)	59 (2)	43 (2)	55 (2)	0.287
Chronic obstructive pulmonary disease	553 (7)	197 (7)	182 (7)	174 (6)	0.514
Cancer	806 (10)	331 (12)	253 (9)	222 (8)	<0.001
Rheumatologic disease	443 (6)	164 (6)	143 (5)	136 (5)	0.248
Chronic kidney disease insufficiency	555 (7)	230 (9)	157 &)	168 (6)	<0.001
Serologic parameters					
Glomerular filtration rate	81.7 (15.0)	80.0 (15.6)	82.2 (14.5)	83.0 (14.7)	<0.01
Albumin (g/dL)	4.6 (0.2)	4.4 (0.2)	4.6 (0.2)	4.7 (0.2)	<0.01
Lymphocytes (n/µL)	1834.1 (617.0)	1407.4 (337.6)	1765.7 (356.5)	2337.9 (679.3)	<0.01
Cholesterol (mmol/L)	219.4 (41.2)	213.2 (40.1)	219.8 (41.0)	225.4 (41.6)	<0.01
Triglycerides (mg/dL)	125.4 (94.8)	113.7 (90.1)	124.4 (94.0)	138.2 (98.7)	<0.01
Medical therapy					
Antihypertensive therapy	3760 (45)	1311 (46)	1208 (44)	1241 (45)	0.150
Antidiabetic therapy	763 (9)	237 (8)	236 (9)	2090 (10)	0.011
Lipid-Lowering therapy	1139 (14)	368 (13)	359 (13)	412 (15)	0.065
Nutritional parameters					
NRI	109.6 (3.7)	106.9 (3.1)	109.9 (2.7)	112.1 (3.3)	<0.01
NRI lowest tertile	2689 (32)	1748 (62)	657 (24)	284 (19)	<0.01
CONUT	0.7 (0.9)	1.3 (1.0)	0.6 (0.7)	0.2 (0.5)	<0.01
CONUT highest tertile	295 (4)	258 (9)	32 (1)	5 (0.2)	<0.01
TCBI	2325.8 (2495.8)	2029.0 (2290.9)	2637.9 (50.2)	2513.0 (47.7)	<0.01
TBI lowest tertile	2796 (33)	1155 (41)	941 (34)	700 (25)	<0.01

Abbreviations: BMI = Body Mass Index; PNI, Prognostic Nutritional Index; NRI, Nutritional Risk Index; CONUT, Controlling Nutritional Status Score; TCBI: Triglyceride × Total Cholesterol × Body Weight Index.

**Table 2:** Univariate and multivariate COX proportional hazards models of nutritional scores for all-cause mortality of the population (*n* = 7789).

		Model 1		Model 2		Model 3	
Nutritional Score	Tertile	HR (95% CI)	*p*-Value	HR (95% CI)	*p*-Value	HR (95% CI)	*p*-Value
PNI	Lowest (<53.3)	2.11 (1.68–2.65)	<0.01	1.60 (1.27–2.01)	<0.01	2.22 (1.61–3.07)	<0.001
	Medium (53.3–56.3)	0.96 (0.74–1.26)	0.70	0.88 (0.68–1.15)	0.37	1.06 (0.74–1.53)	0.74
	Highest (>56.3)	Reference		Reference		Reference	
CONUT	Lowest (0)	Reference		Reference		Reference	
	Medium (1–2)	1.49 (1.23–1.82)	<0.01	1.16 (0.95–1.42)	0.14	1.41 (1.07–1.85)	0.016
	Highest (≥3)	4.67 (3.26–6.12)	<0.01	2.72 (1.97–3.78)	<0.01	2.86 (1.79–4.59)	<0.001
NRI	Lowest (<108.2)	1.59 (1.27–2.00)	<0.01	1.66 (1.32–2.08)	<0.01	2.01 (1.46–2.77)	<0.001
	Medium (108.2–111.2)	1.09 (0.85–1.38)	0.50	1.05 (0.83–1.34)	0.68	1.26 (0.90–1.76)	0.18
	Highest (>111.2)	Reference		Reference		Reference	
TCBI	Lowest (<1336.9)	0.74 (0.59–0.94)	0.12	0.94 (0.74–1.18)	0.57	1.03 (0.75–1.43)	0.84
	Medium (1336.9–2352.4)	0.93 (0.75–1.16)	0.53	0.90 (0.73–1.12)	0.36	1.04 (0.77–1.40)	0.82
	Highest (>2352.4)	Reference		Reference		Reference	

Data are presented as hazard ratios, 95% CI (confidence intervals), and *p*-values. Abbreviations: PNI, Prognostic Nutritional Index; NRI, Nutritional Risk Index; CONUT, Controlling Nutritional Status Score; TCBI: Triglyceride × Total Cholesterol × Body Weight Index.

## Data Availability

The raw data supporting the conclusions of this article will be made available by the authors on request.
